# TRIBE Uncovers the Role of Dis3 in Shaping the Dynamic Transcriptome in Malaria Parasites

**DOI:** 10.3389/fcell.2019.00264

**Published:** 2019-11-01

**Authors:** Meng Liu, Binbin Lu, Yanting Fan, Xiaohui He, Shijun Shen, Cizhong Jiang, Qingfeng Zhang

**Affiliations:** ^1^Institute of Translational Research, Tongji Hospital, Shanghai Key Laboratory of Signaling and Disease Research, The School of Life Sciences and Technology, Tongji University, Shanghai, China; ^2^Research Center for Translational Medicine, Key Laboratory of Arrhythmias of the Ministry of Education of China, East Hospital, Tongji University School of Medicine, Shanghai, China; ^3^The Research Center of Stem Cells and Ageing, Tsingtao Advanced Research Institute, Tongji University, Shanghai, China

**Keywords:** malaria, *Plasmodium falciparum*, TRIBE, Dis3, RIP-seq

## Abstract

Identification of RNA targets of RNA-binding proteins (RBPs) is essential for complete understanding of their biological functions. However, it is still a challenge to identify the biologically relevant targets of RBPs through *in vitro* strategies of RIP-seq, HITS-CLIP, or GoldCLIP due to the potentially high background and complicated manipulation. In malaria parasites, RIP-seq and gene disruption are the few tools available currently for identification of RBP targets. Here, we have adopted the TRIBE (Targets of RNA binding proteins identified by editing) system to *in vivo* identify the RNA targets of PfDis3, a key exoribonuclease subunit of RNA exosome in *Plasmodium falciparum*. We generated a transgenic parasite line of *PfDis3-ADARcd*, which catalyzes an adenosine (A)-to-inosine (I) conversion at the potential interacting sites of PfDis3-targeting RNAs. Most of PfDis3 target genes contain one edit site. The majority of the edit sites detected by PfDis3-TRIBE locate in exons and spread across the entire coding regions. The nucleotides adjacent to the edit sites contain ∼75% of A + T. PfDis3-TRIBE target genes are biases toward higher RIP enrichment, suggesting that PfDis3-TRIBE preferentially detects stronger PfDis3 RIP targets. Collectively, PfDis3-TRIBE is a favorable tool to identify *in vivo* target genes of RBP with high efficiency and reproducibility. Additionally, the PfDis3-targeting genes are involved in stage-related biological processes during the blood-stage development. Thus PfDis3 appears to shape the dynamic transcriptional transcriptome of malaria parasites through post-transcriptional degradation of a variety of unwanted transcripts from both strands in the asexual blood stage.

## Introduction

*Plasmodium falciparum*, a unicellular apicomplexan parasite, causes the most severe clinical outcome of malaria in human. To date, malaria remains a major global health threat with an estimated 400,000 malaria deaths each year worldwide (World Health Organization [WHO], 2018). The pathogenesis of *P. falciparum* in human results from the intra-erythrocytic developmental cycle (IDC), and each step of which is controlled by a precisely timed cascade of gene expression. Throughout the 48-h IDC, a majority of mRNAs reach peak abundance at only one time point, suggesting a strong correlation between transcriptome regulation and pathogenesis (Bozdech et al., 2003).

Recent years, post-transcriptional regulation has emerged as an important pathway in orchestrating biological processes on a transcriptome-wide scale throughout the IDC (Rai et al., 2014; Vembar et al., 2016). Nascent RNA sequencing revealed the pervasive distribution of nascent transcripts in the genome of this parasite, supporting the existence of an overlooked post-transcriptional regulation pathway in shaping the steady-state transcriptome in *P. falciparum* (Lu et al., 2017; Painter et al., 2018). For instance, by an inducible gene knockout strategy, the RNA exosome complex-associated 3′-5′ exoribonuclease subunit, PfDis3, was found to degrade different kinds of antisense lncRANs and a few mRNAs (Droll et al., 2018). Moreover, PfRNase II, an ortholog of Dis3, has been reported to silence a subgroup of the primary virulence genes, *var*, by degrading nascent mRNA *in situ* (Zhang et al., 2014). These studies point to a critical regulatory function of RNA exosome in shaping the transcriptome of malaria parasites by surveillance of various transcripts in the life cycle. However, due to the failure to generate and isolate the pure cells of DiCre recombinase-mediated conditional PfDis3 knockout line, the exact targets and related biological role of PfDis3 in regulating transcriptome of malaria parasites remain to be clarified by other approaches.

Conventional methods to identify *in vivo* targets of RNA-binding proteins (RBP) include CLIP (crosslinking and immunoprecipitation) and variants thereof (Ule et al., 2003, 2005; Corden, 2010; Moore et al., 2014) and RIP (RNA immunoprecipitation) (Gilbert and Svejstrup, 2006). These methods are based on immunoprecipitation with specific antibodies recognizing the RBPs. After covalently binding of RBP to its targets, unprotected RNAs are digested and the remaining RBP-bound RNAs are isolated for high throughput sequencing. These approaches need a high-affinity and specific antibody. The low efficiency of crosslinking step (∼ 1–5%) in CLIP also limits the yield of real targets in IP experiments (Darnell, 2010). It therefore requires large amounts of starting materials (almost millions of cells) and may raise the problem of high false-positive rate which is usually observed in IP experiments. *In P. falciparum*, RIP assay is still the main method to identify targets of RBPs since the CLIP-derived techniques such as PAR-CLIP or GoldCLIP are still not established in this organism so far. To overcome the defects of IP-based methods, McMahon et al. has developed a novel technique termed TRIBE (targets of RNA binding proteins identified by editing) to identify the RNA substrates of RBPs *in vivo*. This system fuses a catalytic domain of the RNA-editing enzyme ADAR (ADARcd) to a RBP of interest and expresses the fusion protein *in vivo* (McMahon et al., 2016). ADAR consists of two double-stranded RNA-binding domains (dsRBDs) and a catalytic domain (ADARcd) that deaminates adenosine to inosine (Bass and Weintraub, 1998; Keegan et al., 2004). By coupling the RBP to only ADARcd, the RBP targets are marked with *de novo* editing events which are identified by RNA sequencing (McMahon et al., 2016). Compared to the methods mentioned above, no immunoprecipitation is needed in TRIBE. Thus, problems like low efficiency of crosslinking and requirement of high affinity, specific antibody or terminal tagging of RBP of interest can be avoided (McMahon et al., 2016). Moreover, in TRIBE assay RNA is simply extracted from cells and sequenced by routine RNA-seq assay. Thus, it requires much less cells than RIP-seq. More importantly, it provides a standard but practice-friendly protocol compared with that of CLIPs (McMahon et al., 2016).

In this study, we sought to adopt the TRIBE technique in *P. falciparum*, by which we were able to identify the substrates of PfDis3 *in vivo*. We have generated *Pfdis3-adar* transgenic parasite line by CRISPR-Cas9 gene editing system, and used it to identify PfDis3-targeted transcripts by TRIBE throughout the IDC in *P. falciparum*. Through detecting *de nova* editing events catalyzed by the PfDis3-ADARcd fusion protein, we found that the majority of the editing sites were located in exonic regions. For the 5602 protein-coding genes in the genome of 3D7 strain, we have identified 2032, 2061 and 2303 genes with editing signals on the sense transcripts, whereas 1522, 2119 and 2187 on the antisense transcripts at ring, trophozoite and schizont, respectively. The TRIBE results were further supported by RIP-seq assay with *Pfdis3-tag* transgenic line by comparative analysis. Moreover, our target genes of PfDis3 were validated by inducible *Pfdis3* gene knockdown analysis. Taken together, by development of TRIBE technique in *P. falciparum*, we reveal that PfDis3 targets are enriched in genes involved in multiple biological processes that are highly relevant to their respective time points of development in IDC, indicating a fundamental function of PfDis3 in surveillance of gene expression throughout the asexual stage in malaria parasites.

## Results

### Generation of Pfdis3-Adar Transgenic Parasite Line by CRISPR-Cas9

**FIGURE 1:**
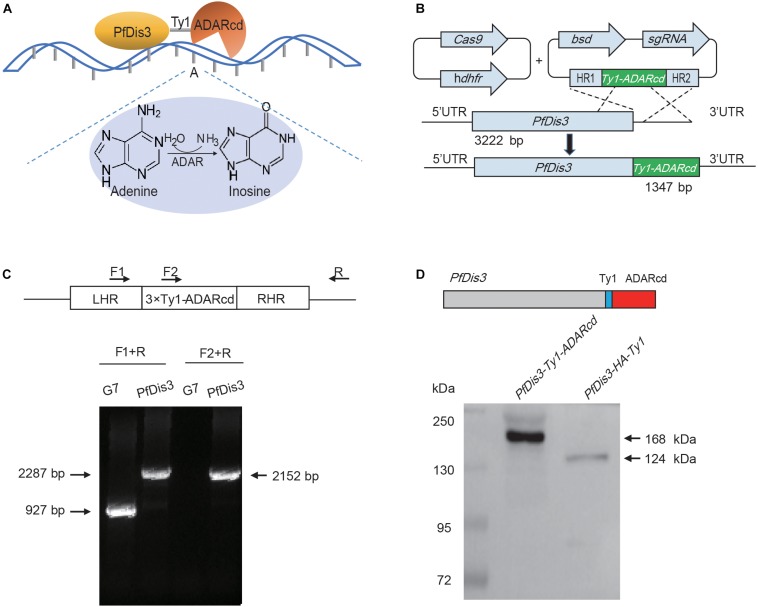
Generation of Pfdis3-adar transgenic parasite line by CRISPR-Cas9. **(A)** PfDis3-ADARcd fusion protein was constructed by attaching a catalytic ADAR protein (ADARcd) to PfDis3. The ADARcd possess a deaminase domain that catalyzes an adenosine-to-inosine conversion. And the editing specificity of the fusion protein is determined by the RNA recognition features of PfDis3. **(B)** Schematic diagram showing the generation of PfDis3-ADARcd transfectant lines by the CRISPR-Cas9 system. The plasmid carrying a single guide RNA (sgRNA) and the Cas9 endonuclease were co-transfected. *bsd*, blasticidin S deaminase. h*dhfr*, human dihydrofolate reductase. HR, homology region. **(C)** qPCR analysis of PfDis3-ADARcd lines. LHR, homology region upstream of Pfdis3-Ty1-ADARcd. RHR, homology region downstream of PfDis3-Ty1-ADARcd. F1, forward primer 1 (5′ gaattgttccatttaagcttttatg 3′). F2, forward primer 2 (5′ cagaagtacatactaaccaagatc 3′). R, reverse primer (5′ acaatgttgttaaattaagtattatg 3′). **(D)** Western blot analysis of PfDis3-ADARcd lines with antibody against Ty1 epitope.

To avoid side effect or growth defect by overexpression of PfDis3-ADARcd, we decided to generate an endogenous integration of the catalytic domain of ADAR enzyme from *Drosophila* into *Pfdis3* gene locus (Figures 1A,B). In order to use CRISPR/Cas9 gene editing technique to achieve fast endogenous integration of the catalytic domain of ADAR enzyme, we constructed the plasmid pL6-PfDis3-Ty1-ADARcd targeting the C-terminus of *Pfdis3* gene and co-transfected with Cas9 expression vector pUF1-cas9 into 3D7 strain (Figure 1B). Approximately 3 weeks after transfection, parasites carrying pL6-PfDis3-Ty1-ADARcd/pUF1-cas9 was obtained by drug selection with both WR99210 (WR) and blasticidin S deaminase (BSD). Next, we cloned this transgenic parasite line and confirmed the integration of Ty1-ADARcd at C-terminus of Pfdis3 gene by PCR (Figure 1C). Western blot assay further confirmed the expression of transgenic ADARcd with the expected molecular weight (MW) (Figure 1D). The specific recognition of the PfDis3-HA-Ty1 fusion protein by antibodies was critical for reducing the background of RIP-seq assay.

TRIBE technology theoretically can detect adenosine (A)-to-inosine (I) editing events on the target transcripts of PfDis3 since the PfDis3-ADARcd protein doesn’t contain the RNA recognition domain of ADAR (Figure 1A). To eliminate the background due to genomic mutation or Single Nucleotide Polymorphism (SNPs), we have re-sequenced the genome sequence of wild-type 3D7-G7 clone for transfection of *PfDis3-ADARcd* construct. Next, by strand-specific RNA sequencing of PfDis3-ADARcd transgenic parasite clone, we were able to identify the *de novo* editing events on the target transcripts of PfDis3 on individual strands of chromosomes. The wild-type 3D7-G7 clone was used as control to exclude endogenous RNA editing events in the parasites. To comprehensively identify the targets of Pfdis3, we harvested the synchronized parasites at ring (R), trophozoite (T) and schizont (S) asexual developmental stages, respectively. Meanwhile, the PfDis3-HA-Ty1 tagging line was collected in parallel for RIP-seq analysis with specific antibody against Ty1 epitope.

### PfDis3-ADARcd Edits Evenly Along the Transcripts From 5′UTR to 3′UTR

By nucleotide sequence-based stranded comparative transcriptome analysis between PfDis3-ADARcd and WT 3D7-G7 clone, a total of 3643, 3626, 3387 editing sites on the sense transcripts and 2671, 3765, 3789 editing sites on the antisense transcripts were detected for the three asexual developmental stages (R, T, S), respectively. These editing sites correspond to 2032, 2061, 2303 genes and 1522, 2119, 2187 antisense non-coding RNAs, respectively. Besides the known structured RNAs processed by PfDis3 protein such as rRNAs and small nucleolar RNAs, we also identified many PfDis3 targets of various functions corresponding to their respective developing stages (Supplementary Table S1).

**FIGURE 2:**
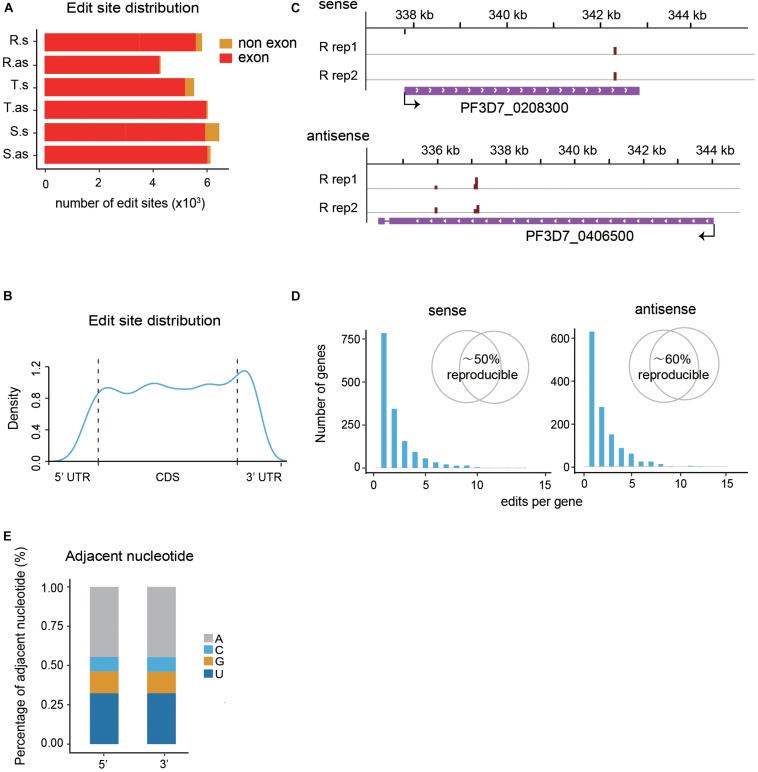
Profiles of PfDis3-ADARcd editing events during the IDC in *P. falciparum*. **(A)** Boxplots showing the numbers of editing events detected in exons and non-exonic regions for sense (s) and antisense (as) transcripts during the IDC, respectively. R.s, T.s, and S.s are sense transcripts at Ring, Trophozoite and Schizont stages. R.as, T.as, and S.as are antisense transcripts at Ring, Trophozoite and Schizont stages. **(B)** Track view of editing events in two representative genes, PF3D7_0208300 (sense) and PF3D7_0406500 (antisense). The data are from the two biological replicates at R stage. The red bars indicate the editing events. The height of the bar indicates the editing ratio in that site. **(C)** Distribution of edit sites along transcripts. **(D)** Frequency histograms indicate the number of edit sites per target transcript for sense and antisense transcripts. The inset Venn diagram showing the percentage of target genes detected in both replicates. **(E)** Barplot showing the sequence composition of the 5′ and 3′ nucleotides adjacent to the PfDis3-ADARcd edit sites.

The majority of the *de novo* edit sites of PfDis3-ADARcd locate in exons (Figure 2A). Moreover, the edit sites are evenly distributed along the transcripts with minor enrichment in the 3′UTR (Figures 2B,C). This enrichment feature is consistent with Dis3’s catalytic feature as endonuclease and exonuclease (Lebreton et al., 2008; Schaeffer et al., 2009; Schneider et al., 2009). The number of the edit sites in each transcript ranges from 1 to 15. Most of transcripts contain single edit site (Figure 2D). Interestingly, the editing events are highly reproducible both in position and efficiency (Figure 2D, inset and Supplementary Figure S1A). The median percentage editing levels of *de novo* editing events in both sense and antisense transcripts during the IDC are about 8% (Supplementary Figure S1B). To explore whether the number of editing sites in each gene would infect editing ratio, we grouped transcripts by their total number of editing sites and compared their editing ratio among different groups. The results show no obvious correlation between the number of editing sites and editing ratio for both sense and antisense transcripts (Supplementary Figure S1C).

It has been reported previously that the *P. falciparum* genome has the highest AT composition among all the organisms sequenced to date (Gardner et al., 2002). Consequently, the mRNA transcriptome possesses higher level of adenosine in *P. falciparum* than other organisms. We wondered whether there is sequence composition bias in flanking sequences of the editing sites. To this end, we examined the adjacent nucleotides of editing sites. Unlike the former study that ADAR preferred an editing sequence of UAG (Rahman et al., 2018), the results show that the most frequent adjacent nucleotide is A, the second T, the third G, then C (Figure 2E). The special editing environment of PfDis3-TRIBE may reflect the unique composition of *P. falciparum* genome.

### TRIBE Exhibits Higher Sensitivity and Reproducibility in Identification of PfDis3 Targets in *P. falciparum*

**FIGURE 3:**
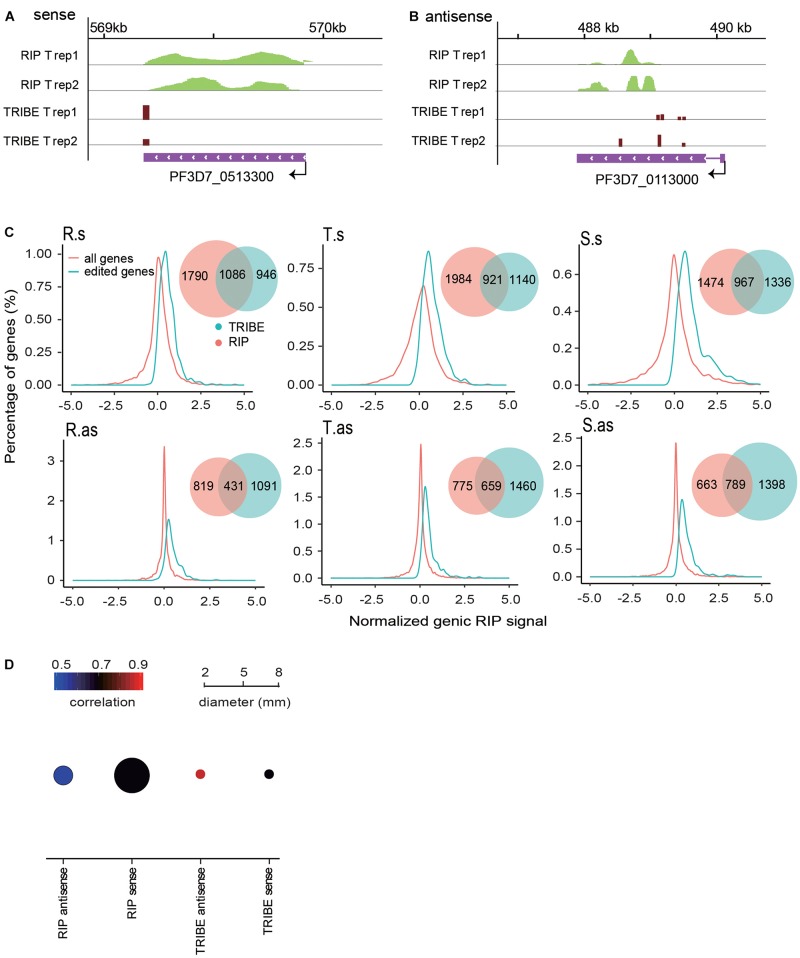
PfDis3-TRIBE identifies targets with higher sensitivity and reproducibility than PfDis3-RIP in *P. falciparum*. **(A,B)** Track view showing RIP signals and editing events in the two representative target genes, PF3D7_0513300 (a, sense) and PF3D7_0113000 (b, antisense). The data are from the two biological replicates of RIP and TRIBE, respectively. **(C)** Distribution of normalized genic RIP signal in all RIP target genes (red) and PfDis3-ADARcd edited genes (green). The inset Venn plot shows the overlap of the target genes identified by both PfDis3-TRIBE (green) and PfDis3-RIP (red). R.s, T.s, and S.s are sense transcripts at Ring, Trophozoite and Schizont stages. R.as, T.as, and S.as are antisense transcripts at Ring, Trophozoite and Schizont stages. **(D)** Bubble plot showing the Pearson correlations between the replicates for PfDis3-TRIBE and PfDis3-RIP at ring (R), trophozoite (T) and schizont (S) stages, respectively. The color indicates the mean value of correlation coefficients. The size of the circle indicates the variance of correlation coefficients.

To further assess the reliability of our editing result, we performed a series of strand-specific RIP-seq experiments with PfDis3-HA-Ty1 parasite line to identify the potential targets of PfDis3. The RIP enrichment ratio relative to the expression level of transcripts was calculated and normalized. We observed a high correlation of RIP signals in the biological replicates (Supplementary Figure S2). The targets identified by RIP are also reproducible (Supplementary Figure S2, Venn diagram). Due to the low resolution, RIP signals are broad and spread across the gene body (Figures 3A,B). Interestingly, TRIBE detected editing sites in the genes with RIP signals in the gene body. Notably, TRIBE detected editing sites with 1-bp resolution. Surprisingly, the target genes identified by TRIBE are biased toward higher RIP signals when compared to the target genes identified by RIP (Figure 3C). Indeed, 36, 39, and 47% of RIP targets are also TRIBE targets at R, T and S stages, respectively (Figure 3C, Venn plot). Consistently, it has been reported previously that TRIBE was able to detect high-confidence CLIP targets (McMahon et al., 2016). Moreover, the correlation coefficient between replicates in TRIBE assay is higher and has a much smaller variation than that of RIP assay for either sense or antisense targets (Figure 3D and Supplementary Figures S1A, S2). Collectively, these findings suggest that TRIBE can reliably identify the targets of PfDis3 with high resolution in *P. falciparum*.

### PfDis3 Regulates the Dynamical Transcriptional Program During the Asexual Blood Stage in *P. falciparum*

**FIGURE 4:**
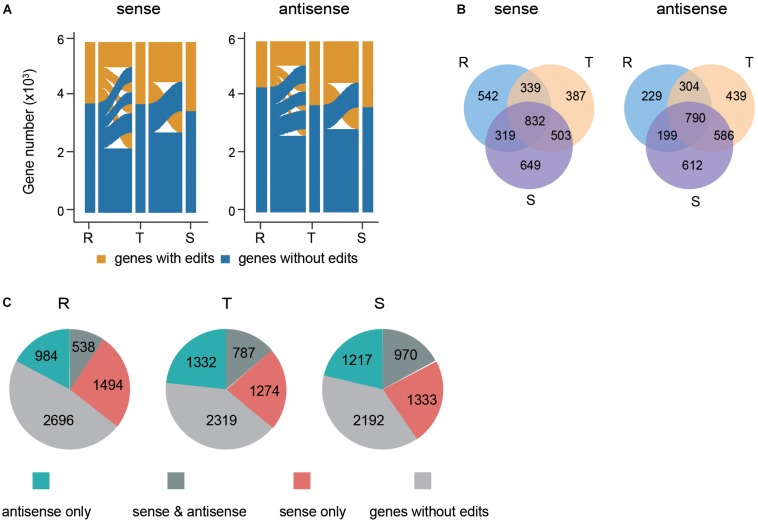
Dynamics and features of PfDis3-TRIBE target genes during the IDC in *P. falciparum*. **(A)** Dynamic change of editing events in sense transcripts and antisense transcripts during the IDC. R, ring. T, trophozoite. S, schizont. **(B)** Overlap of PfDis3-TRIBE sense and antisense target transcripts at ring (R), trophozoite (T) and schizont (S) stages. **(C)** Pie chart showing the number of categorized target genes detected by TRIBE at ring (R), trophozoite (T) and schizont (S) stages, respectively. sense only, Pfdis3 binds to the sense transcripts but not the antisense transcripts of the genes. antisense only, Pfdis3 binds to the antisense transcripts but not the sense transcripts of the genes. sense and antisense, Pfdis3 binds to both antisense and sense transcripts of the genes. Genes without edits, genes with no editing events detected.

We next investigated the dynamics of PfDis3 targets during the IDC. The results show that there are no editing sites in ∼ one third of genes across the IDC whereas one sixth of genes persistently contain editing sites. The rest of transcripts dynamically contain editing sites (Figure 4A). The common targets of PfDis3 across the IDC account for 41, 40, and 36% of sense targets and 52, 37, and 36% of antisense targets for R, T and S stages, respectively. In contrast, only 27, 19, and 28% of sense targets and 15, 21, and 28% of antisense targets are specific to R, T and S stages, respectively (Figure 4B). This indicates that the targets of PfDis3 only show stage specificity to a certain extent. We further examined the correlation between the sense targets and antisense targets of PfDis3 at R, T and S stages, respectively. The results show that 26, 38, and 42% of sense targets contain edit site(s) in the corresponding antisense targets for R, T and S stages, respectively. Similarly, 35, 37, and 44% of antisense targets contain edit site(s) in the corresponding sense targets for R, T and S stages, respectively (Figure 4C).

**FIGURE 5:**
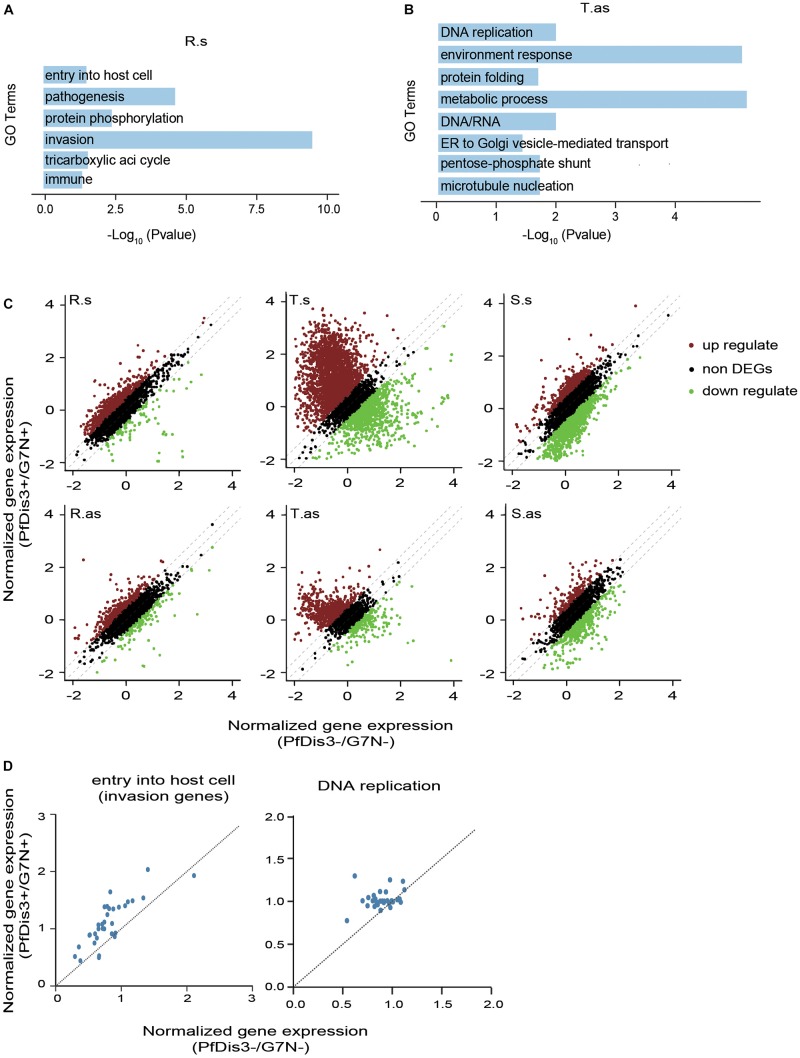
The functions of PfDis3-TRIBE target genes during the IDC in *P. falciparum*. **(A)** Enriched Gene Ontology (biological processes) terms for PfDis3-TRIBE sense target genes at ring stage. **(B)** Enriched GO terms for PfDis3-TRIBE antisense target genes at trophozoite stage. **(C)** Scatter plots showing expression levels of sense (top) and antisense (bottom) transcripts at R, T and S stages with (*Y* axis) and without (*X* axis) Pfdis3 knock down. Transcripts with fold change ≥1.35 are differentially expressed transcripts. **(D)** Scatter plots showing that the majority of the genes in the GO term “entry into host cell” are up-regulated at R stage upon Pfdis3 knockdown (left), and that the majority of the genes in the GO term “DNA replication” are up-regulated at T stage upon Pfdis3 knockdown (right).

To understand the functions of the target genes of PfDis3, we performed Gene Ontology (GO) analysis of the target genes. The results show that the target genes at R, T and S stages are enriched in the stage-related functions (Figures 5A,B and Supplementary Figures S3A,B). For example, the sense target genes at R stage are enriched for entry into host cell, pathogenesis, invasion, etc. (Figure 5A). This is consistent with infection and inhabitation of the parasites in red cells at R stage. In contrast, the antisense target genes at T stage are enriched for DNA replication, protein folding, metabolic process, etc. (Figure 5B). This is consistent with proliferation of the parasites at T stage.

We next constructed conditional Pfdis3 knock down (KD) parasite lines PfDis3-DD with ribozyme system (Droll et al., 2018) to investigate how PfDis3 impacts gene transcription. We used RNA-seq to measure gene expression levels that are highly reproducible (Supplementary Figure S3C). After Pfdis3 knockdown by drug induction, a number of genes are significantly differentially expressed. Especially, there are much more significantly up-regulated genes than down-regulated genes at R and T stages (Figure 5C). Intriguingly, the majority of genes in the GO term “entry into host cell” are up-regulated at R stage upon Pfdis3 knockdown. Similarly, the majority of genes in the GO term “DNA replication” are up-regulated at T stage upon Pfdis3 knockdown (Figure 5D). This is consistent with the functions of the target genes (Figures 5A,B).

## Discussion

The RNA exosome complex is highly conserved in eukaryotic organisms. In *P. falciparum*, the RNA exosome is composed of seven distinct core exosome subunits and two canonical 3′-5′ exoribonuclease factors: PfDis3 and PfRrp6 (Droll et al., 2018). For Dis3 protein, it harbors both processive exonucleolytic and endonucleolytic activities originating from the RNB and PIN domains, respectively (Lebreton et al., 2008; Schaeffer et al., 2009; Schneider et al., 2009). In yeast, Dis3 is present in both the nucleus and cytoplasm (Gudipati et al., 2012; Schneider et al., 2012) and human Dis3 proteins are differently localized (Staals et al., 2010; Tomecki et al., 2010; Lubas et al., 2013). Disruption of human dis3 (hRrp44) gene uncovered the cryptic transcription upstream of active human promoters (Preker et al., 2008). By combining the transcriptomic analysis of human cell with dis3 gene mutation and Photoactivatable Ribonucleoside-Enhanced Cross-Linking and Immunoprecipitation (PAR-CLIP) assay, the RNA polymerase II transcriptome in humans was found to be controlled by Dis3 through decay of unwanted transcripts associated with ∼50% of transcribed protein-coding genes and unannotated genomic regions covering ∼70% of the genome. In *P. falciprum*, PfDis3 was detected predominately in the cytoplasmic fraction during asexual blood stage, but the majority of target transcripts of PfDis3 were antisense lncRNAs measured by DiCre knockout and RNA-seq (Droll et al., 2018). Here, by using TRIBE analysis, we uncover that PfDis3 is a global post-transcriptional regulator of protein coding and non-coding transcripts over the course of blood-stage development of the parasites. The function of PfDis3 in shaping cellular transcriptome is likely evolutionary conserved in eukaryotes from *Plasmodium* to human.

The human malaria parasite *P. falciparum* has a special nucleotide composition of genome with extremely higher AT content compared to other organisms (Gardner et al., 2002). Moreover, the mRNA transcriptome displays even stronger adenosines bias of about 45% (Baumgarten et al., 2019). The special sequence composition and other reasons make it hard to identify RBP targets in *P. falciparum* with traditional immunoprecipitation-based methods. In CLIP, crosslinking needs to be performed beforehand, raising a bias of preferential crosslinking of uredines (Fecko et al., 2007). It has not been reported any successful CLIP experiment in *P. falciparum* to date. RIP also faces problems like choice of RNase and the fragmentation condition that has a significant impact on the detected targets (Lambert et al., 2014). Many RIP results are not reproducible due to the non-specific binding of antibody in *P. falciparum*. To circumvent these issues, we adopted PfDis3-TRIBE in *P. falciparum* to identify Pfdis3 targets throughout IDC for the first time. We first identified Pfdis3 targeted sense/antisense transcripts at ring, trophozoite and schizont stage, respectively (Supplementary Table S1). We found that many Pfdis3 targets were antisense transcripts. Both sense and antisense Pfdis3 targets were enriched in biological processes that are highly relevant for their developmental stages, indicating that Pfdis3 dynamically regulates the transcriptional program of *P. falciparum* throughout the IDC and maintains the steady state level of transcriptome.

We also performed RIP experiment and conditional Pfdis3 knockdown (KD) to evaluate the reliability of the target genes identified by PfDis3-TRIBE. PfDis3-TRIBE target genes were biased toward higher RIP enrichment, indicating that PfDis3-TRIBE preferentially detects stronger RIP-seq targets (Figure 3C). Moreover, most of the identified Pfdis3 target genes across the IDC were upregulated upon Pfdis3 KD (Figure 5C). Taken together, all these results suggest that PfDis3-TRIBE is able to identify Pfdis3 targets with higher confidence and better reproducibility than RIP.

## Materials and Methods

### Plasmid Construction

To generate plasmid *Pfdis3-Ty1-ADARcd and Pfdis3-Ty1-ribo* for transfection, we modified the plasmid *pL6-gfp* by replacing the *gfp* box with a ∼1-kb homolog sequence flanking the N- or C-terminus of the target genes which contained three copies of Ty1epitope and ADARcd or *glmS* ribozyme gene, and inserting a guide RNA sequence specific to the *Pfdis3* gene (PF3D7_1359300) by In-Fusion PCR Cloning System, respectively (Supplementary Table S2). The resulting plasmids were *pL6-Pfdis3-Ty1-ADARcd* and *pL6-Pfdis3-Ty1-Ribo*. The plasmid pUF1-Cas9-infusion carrying Cas9 expression cassette was modified by replacing the original y*dhodh* gene with h*dhfr*.

### Parasite Culture and Transfection

*Plasmodium falciparum* parasites were cultured in human red blood cell in culture medium (10.44 g/L RPMI-1640, 25 mM HEPES, 10% v/v Albumax I, 0.1 mM hypoxanthine, 20 μg/ml gentamicin) under 5% O_2_, 3% CO_2_ at 37°C. For synchronization, ring-stage parasites were enriched with 5% sorbitol, late-stage parasites were enriched via 40 and 70% percoll. Fresh red blood cell were electroporated with 100 μg of plasmid sgRNA and Cas9 in cytomix (120 mM KCl, 2 mM EGTA, 10 mM KH_2_PO_4_, 25 mM HEPES pH7.8, 0.15 mM CaCl_2_, 5 mM MgCl_2_) and then the synchronization of late-stage parasites were added. After 2 or 3 cycles, transfected parasites were selected by WR99210 and blasticidin S deaminase drug. The integration DNA was validated by PCR following sequencing and the proteins were identified by Western blot.

### Western Blot

Total proteins were extracted with 0.15% saponin, washed with cold PBS (pH 7.4) until the supernatant was clear and then resuspended in 1 × SDS-loading buffer following heating under 100°C for 5 min. Proteins were separated by gel electrophoresis and transferred to a PVDF membrane. The membrane was blocked with 5% milk, incubated with primary antibody, washed with PBST, and then incubated with secondary antibody which could generate a chemiluminescent signal. The proteins were visualized by exposing to an imaging device. The materials used in this study were mouse anti-PfAldolase (1:1000, Abcam), mouse anti-Ty1 (1:500, Sigma), ECL Western blotting kit (GE healthcare) (Freeman, 2013).

### RNA Extraction and Stranded RNA-Seq

Red blood cells infected by highly synchronous parasites were collected by centrifugation and resuspended in Trizol that could be stored in −80°C for a long time. After centrifugation, the supernatant was saved and then RNA was extracted according to reagent specification of Direct-zol^TM^ RNA MiniPrep (R2052). The integrality of RNA was validated by 2% agarose gel. Library construction was performed based on KAPA Stranded mRNA-Seq Kit (KK8421).

### RIP-Seq

Infected red blood cells (iRBCs) were collected by centrifugation and resuspended in cold PBS (10 volume of iRBC). Parasites were extracted with 0.15% saponin and washed with cold PBS until the supernatant was clear. Lysis buffer (50 mM Tris-Cl pH7.4, 150 mM NaCl, 1 mM EDTA, 1 mM EGTA, 1% Triton X-100/NP-40) with protease inhibitors and RNase inhibitors was added to the parasite pellet [1.5 ml/(1 × 10^9^ parasites)] and incubated with rotation for 1 h at 4°C. Cell debris were spun out at 12000 × *g* for 15 min at 4°C and supernatant was incubated with 10 μg of anti-Ty1 antibodies at 4°C for 3 h with rotation. For preparation of equilibrated protein-G magnetic beads, 50 μl of protein-G magnetic beads were washed once with Wash Buffer (10 mM Tris-Cl pH 7.5, 150 mM MgCl_2_, 150 mM KCl, 0.1% Triton X-100), then washed once with Elution Buffer, and washed twice with Wash Buffer. Protein-G magnetic beads were mixed with supernatant with protease inhibitors and RNase inhibitors at 4°C overnight with rotation. Beads were washed twice with 500 μl of Wash Buffer and once with PBS and then resuspended in 700 μl of Trizol at 4°C for 10 min. After removal of the beads, 140 μl of chloroform was added to Trizol at room temperature for 5 min and mixture was centrifuged at 12000 × *g* for 30 min at 4°C. Supernatant was added to a new 1.5 ml centrifuge tube and centrifuged at 12000 × *g* for 2 min at 4°C. Supernatant was mixed with 1 μl of glycogen and equal volume of isopropanol on ice for 2 h. Tube was centrifuged at 12000 × *g* for 30 min at 4°C. Pellet was washed twice with 1 ml of 75% (vol/vol) ethanol, then air-dried and resuspended in 20 μl of RNase-free water (Chomczynski and Sacchi, 2006). DNase I was used to digest DNA for 15 min at room temperature. RNase-free water was added up to 100 μl, mixed with equal volume of chloroform/isoamyl alcohol pH < 5.0 (24:1), and then mixture was centrifuged at 12000 × *g* for 5 min. Supernatant was mixed with phenol/chloroform/isoamyl alcohol (25:24:1), and centrifuged at 12000× *g* for 5 min. Supernatant was transferred to a new 1.5 ml centrifuge tube with 1 μl of glycogen, one-tenth volumes of 3 M sodium acetate (pH 5.2), and 2.5 volumes of 100% ethanol and incubated at -80°C for 20 min. Samples were centrifuged at 12000× *g* for 15 min and pellet was washed twice with 1 ml of 75% (vol/vol) ethanol, then air-dried and resuspended in 11 μl of RNase-free water. Construction library was performed using KAPA Stranded mRNA-Seq Kit (KK8421).

### RIP Sequencing Analysis

RIP sequencing reads were trimmed with cutadapt (v11) by 10 bp at each end. Reads with average quality score ≥20 and length ≥50 bp were retained. The strand specific reads were aligned with hisat2 (v2.1.0) (Kim et al., 2015) (parameters: –rna-strandness RF –dta –no-discordant –no-mixed –no-unal). Read counts for sense/antisense transcripts were calculated using featureCounts (v1.6.1) with parameters: -M -p -B -C for all; -s 2 for sense transcripts; -s 1 for antisense transcripts (Liao et al., 2014). Both sense and antisense read counts were merged for library normalization between conditions. The final expression levels of sense/antisense transcripts were normalized as FPKM (Fragment Per Kilobase of transcript per Million reads mapped) for further analysis. RIP-seq (i.e., PfDis3-RIP and GFP-RIP) data were normalized with the transcript abundance of their corresponding time point. We also did quantile normalization for former normalized RIP data to make it comparable among samples. Only transcripts with minimum 1.2 fold of PfDis3-RIP versus GFP-RIP were considered as PfDis3-RIP enriched transcripts. As biological replicates of the same treatment and developmental stage were highly correlated (Supplementary Figure S2), we combined them for the downstream analysis.

### Analysis of Sequencing Data for RNA Editing

RNA editing data was analyzed as the previously published manual (McMahon et al., 2016) with modifications. In brief, pair-end sequencing reads were trimmed with cutadapt (v11) by 10 bp at each end, reads with average quality score ≥20 and length ≥50 bp were retained. Genomic DNA reads were aligned to the *P. falciparum* genome (plasmoDB.org, v3 release 32) (Gardner et al., 2002) using bwa (v 0.7.17-r1188) (Li and Durbin, 2009, 2010; Li, 2012) with default parameter. Strand specific RNA sequencing reads were aligned using Tophat2 (v2.1.1) (Kim et al., 2013) (parameters: -m 1 –g 2 -I 50000 –microexon-search –no-coverage-search –library-type fr-firststrand). PCR duplicates were removed using sambamba (v0.6.6) (Tarasov et al., 2015) for editing analysis and reads were sorted using sambamba (v0.6.6). We next converted the sorted files to a matrix using a previously published perl script sam_to_matrix.pl (McMahon et al., 2016). Genomic nucleotide information and transcript nucleotide information were merged using bedtools (v2.27.1) (Quinlan and Hall, 2010) for further identification of edit sites.

Only sites with minimum 10 coverage and 5% editing ratio were considered to be valid edit sites. gDNA coverage ≥30 and uniformity of nucleotide were also required to avoid SNP. The editing ratios are highly reproducible for the biological replicates (Supplementary Figure S1A), therefore, we combined the biological replicates for the downstream analysis. RNA editing data was converted to bedgraph file format for display using R (v3.5.1).

### RNA-Seq Data Analysis

Illumina adapters were removed with cutadapt (v11), reads with average quality score ≥20 and length ≥50 bp were retained. RNA sequencing reads were aligned using hisat2 (v2.1.0) (Kim et al., 2015) with strand specific mode (i.e., –rna-strandness RF). Read counts for sense/antisense transcripts were obtained using featureCounts (v1.6.1) (Liao et al., 2014). Both sense and antisense read counts were merged for library normalization between conditions. FPKM of sense/antisense transcripts were calculated using R (v3.5.1). The gene expression levels were highly reproducible for the biological replicates of the same treatment and developmental stage (Supplementary Figure S3C), therefore, we used the replicate one for further analysis. Genes with minimum 1.35 fold (Pfdi3 KD versus G7) were considered as differentially expressed genes between two samples.

### Gene Ontology Analysis

Gene ontology (GO) enrichment was performed using R (v3.5.1). GO terms database was downloaded from Plasmodb^1^. GO terms with *p*-value ≥0.05 (Fisher exact test) and enriched gene number ≥5 were considered to be enriched. We next classified our GO terms into different functional categories for better understanding.

## Data Availability Statement

The raw sequence data reported in this paper have been deposited in the Gene Expression Omnibus (GEO) under the accession number: GSE133654.

## Author Contributions

QZ and CJ conceived and designed the experiments. YF and XH generated transgenic parasite lines of PfDis3-ADARcd and PfDis3-DD. BL performed WB, TRIBE, and RNA-seq assay. ML and SS performed informatics analysis. QZ, CJ, and ML wrote the manuscript. All authors read and approved the final manuscript.

## Conflict of Interest

The authors declare that the research was conducted in the absence of any commercial or financial relationships that could be construed as a potential conflict of interest.

## Footnotes

1 https://plasmodb.org/plasmo/

## Supplementary Material

FIGURE S1 The PfDis3-ADARcd reproducibly edits certain sites in *P.falciparum* during the IDC. **(A)** The edit sites at sense (top) transcripts and antisense (bottom) transcripts are reproducible in editing frequency at ring (R), trophozoite (T) and schizont (S) stages. Pearson correlation between biological replicates are shown. **(B)** Box plot showing the PfDis3-ADARcd editing levels in sense (s) transcripts and antisense (as) transcripts at ring (R), trophozoite (T) and schizont (S) stages. **(C)** Box plots showing the editing frequency of sense transcripts and antisense transcripts grouped by the number of edit sites. The middle line in the box indicates the mean of the editing frequency in the transcripts with more than one edit sites.

FIGURE S2 Reproducibility of PfDis3-RIP assay across developmental stages. Correlation of genic RIP signals in sense (s) transcripts and antisense (as) transcripts between biological replicates. Pearson correlation coefficients between biological replicates are displayed at top left corner. The inset Venn diagram showing the overlap of PfDis3 targets identified by PfDis3-RIP between the two replicates. R, T, S indicates Ring, Trophozoite and Schizont stage, respectively.

FIGURE S3 Functions of PfDis3-TRIBE target genes during the IDC in *P. falciparum*. **(A)** Enriched Gene Ontology (biological processes) terms for PfDis3 sense target transcripts at T (left panel) and S (right panel) stages, respectively. **(B)** Enriched GO terms for PfDis3 antisense target transcripts at ring R (left panel) and S (right panel) stages, respectively. **(C)** Correlation for expression of sense (top) transcripts and antisense (bottom) transcripts between biological replicates. Pearson correlation coefficients between biological replicates are displayed at top left corner. R, T and S indicates Ring, Trophozoite, Schizont stage, respectively.

TABLE S1 List of sense/antisense target genes identified by PfDis3-TRIBE during the IDC in *P. falciparum*.

TABLE S2 Primers and homology sequences for our constructed parasites.

## References

[B1] Bass B. L. Weintraub H. (1998). An unwinding activity that covalently modifies its double-stranded RNA substrate. *Cell* 55:10. 3203381 10.1016/0092-8674(88)90253-x

[B2] Baumgarten S. Bryant J. M. Sinha A. Reyser T. Preiser P. R. Dedon P. C. (2019). Transcriptome-wide dynamics of extensive m(6)A mRNA methylation during *Plasmodium falciparum* blood-stage development. *Nat. Microbiol.* 10.1038/s41564-019-0521-7 [Epub ahead of print]. 31384004 PMC7611496

[B3] Bozdech Z. Llinas M. Pulliam B. L. Wong E. D. Zhu J. C. Derisi J. L. (2003). The transcriptome of the intraerythrocytic developmental cycle of *Plasmodium falciparum*. *PLoS Biol.* 1:085. 10.1371/journal.pbio.0000005 12929205 PMC176545

[B4] Chomczynski P. Sacchi N. (2006). The single-step method of RNA isolation by acid guanidinium thiocyanate-phenol-chloroform extraction: twenty-something years on. *Nat. Protoc.* 1 581–585. 10.1038/nprot.2006.83 17406285

[B5] Corden J. L. (2010). Shining a new light on RNA-protein interactions. *Chem. Biol.* 17 316–318. 10.1016/j.chembiol.2010.04.003 20416501

[B6] Darnell R. B. (2010). HITS-CLIP: panoramic views of protein-RNA regulation in living cells. *Wiley Interdiscip. Rev. RNA* 1 266–286. 10.1002/wrna.31 21935890 PMC3222227

[B7] Droll D. Wei G. Guo G. Fan Y. Baumgarten S. Zhou Y. (2018). Disruption of the RNA exosome reveals the hidden face of the malaria parasite transcriptome. *RNA Biol.* 15 1206–1214. 10.1080/15476286.2018.1517014 30235972 PMC7000224

[B8] Fecko C. J. Munson K. M. Saunders A. Sun G. Begley T. P. Lis J. T. (2007). Comparison of femtosecond laser and continuous wave UV sources for protein-nucleic acid crosslinking. *Photochem. Photobiol.* 83:1394. 10.1111/j.1751-1097.2007.00179.x 18028214

[B9] Freeman L. A. (2013). Western blots. *Methods Mol. Biol.* 1027 369–385. 10.1007/978-1-60327-369-5_18 23912997

[B10] Gardner M. J. Hall N. Fung E. White O. Berriman M. Hyman R. W. (2002). Genome sequence of the human malaria parasite *Plasmodium falciparum*. *Nature* 419 498–511. 12368864 10.1038/nature01097 PMC3836256

[B11] Gilbert C. Svejstrup J. Q. (2006). RNA immunoprecipitation for determining RNA-protein associations in vivo. *Curr. Protoc. Mol. Biol.* 27:11. 10.1002/0471142727.mb2704s75 18265380

[B12] Gudipati R. K. Xu Z. Y. Lebreton A. Seraphin B. Steinmetz L. M. Jacquier A. (2012). Extensive degradation of RNA precursors by the exosome in wild-type cells. *Mol. Cell.* 48 409–421. 10.1016/j.molcel.2012.08.018 23000176 PMC3496076

[B13] Keegan L. P. Leroy A. Sproul D. O’connell M. A. (2004). Adenosine deaminases acting on RNA (ADARs): RNA-editing enzymes. *Genome Biol.* 5:209. 14759252 10.1186/gb-2004-5-2-209 PMC395743

[B14] Kim D. Langmead B. Salzberg S. L. (2015). HISAT: a fast spliced aligner with low memory requirements. *Nat. Methods* 12 357–360. 10.1038/nmeth.3317 25751142 PMC4655817

[B15] Kim D. Pertea G. Trapnell C. Pimentel H. Kelley R. Salzberg S. L. (2013). TopHat2: accurate alignment of transcriptomes in the presence of insertions, deletions and gene fusions. *Genome Biol.* 14:R36. 10.1186/gb-2013-14-4-r36 23618408 PMC4053844

[B16] Lambert N. Robertson A. Jangi M. Mcgeary S. Sharp P. A. Burge C. B. (2014). RNA Bind-n-Seq: quantitative assessment of the sequence and structural binding specificity of RNA binding proteins. *Mol. Cell.* 54 887–900. 10.1016/j.molcel.2014.04.016 24837674 PMC4142047

[B17] Lebreton A. Tomecki R. Dziembowski A. Seraphin B. (2008). Endonucleolytic RNA cleavage by a eukaryotic exosome. *Nature* 456 993–996. 10.1038/nature07480 19060886

[B18] Li H. (2012). Exploring single-sample SNP and INDEL calling with whole-genome de novo assembly. *Bioinformatics* 28 1838–1844. 10.1093/bioinformatics/bts280 22569178 PMC3389770

[B19] Li H. Durbin R. (2009). Fast and accurate short read alignment with burrows-wheeler transform. *Bioinformatics* 25 1754–1760. 10.1093/bioinformatics/btp324 19451168 PMC2705234

[B20] Li H. Durbin R. (2010). Fast and accurate long-read alignment with burrows-wheeler transform. *Bioinformatics* 26 589–595. 10.1093/bioinformatics/btp698 20080505 PMC2828108

[B21] Liao Y. Smyth G. K. Shi W. (2014). featureCounts: an efficient general purpose program for assigning sequence reads to genomic features. *Bioinformatics* 30 923–930. 10.1093/bioinformatics/btt656 24227677

[B22] Lu X. M. Batugedara G. Lee M. Prudhomme J. Bunnik E. M. Le Roch K. G. (2017). Nascent RNA sequencing reveals mechanisms of gene regulation in the human malaria parasite *Plasmodium falciparum*. *Nucleic Acids Res.* 45 7825–7840. 10.1093/nar/gkx464 28531310 PMC5737683

[B23] Lubas M. Damgaard C. K. Tomecki R. Cysewski D. Jensen T. H. Dziembowski A. (2013). Exonuclease hDIS3L2 specifies an exosome-independent 3′-5′ degradation pathway of human cytoplasmic mRNA. *EMBO J.* 32 1855–1868. 10.1038/emboj.2013.135 23756462 PMC3981170

[B24] McMahon A. C. Rahman R. Jin H. Shen J. L. Fieldsend A. Luo W. (2016). TRIBE: hijacking an RNA-editing enzyme to identify cell-specific targets of RNA-binding proteins. *Cell* 165 742–753. 10.1016/j.cell.2016.03.007 27040499 PMC5027142

[B25] Moore M. J. Zhang C. Gantman E. C. Mele A. Darnell J. C. Darnell R. B. (2014). Mapping argonaute and conventional RNA-binding protein interactions with RNA at single-nucleotide resolution using HITS-CLIP and CIMS analysis. *Nat. Protoc.* 9 263–293. 10.1038/nprot.2014.012 24407355 PMC4156013

[B26] Painter H. J. Chung N. C. Sebastian A. Albert I. Storey J. D. Llinas M. (2018). Genome-wide real-time in vivo transcriptional dynamics during *Plasmodium falciparum* blood-stage development. *Nat. Commun.* 9:2656. 10.1038/s41467-018-04966-3 29985403 PMC6037754

[B27] Preker P. Nielsen J. Kammler S. Lykke-Andersen S. Christensen M. S. Mapendano C. K. (2008). RNA exosome depletion reveals transcription upstream of active human promoters. *Science* 322 1851–1854. 10.1126/science.1164096 19056938

[B28] Quinlan A. R. Hall I. M. (2010). BEDTools: a flexible suite of utilities for comparing genomic features. *Bioinformatics* 26 841–842. 10.1093/bioinformatics/btq033 20110278 PMC2832824

[B29] Rahman R. Xu W. Jin H. Rosbash M. (2018). Identification of RNA-binding protein targets with HyperTRIBE. *Nat. Protoc.* 13 1829–1849. 10.1038/s41596-018-0020-y 30013039 PMC6349038

[B30] Rai R. Zhu L. Chen H. F. Gupta A. P. Sze S. K. Zheng J. (2014). Genome-wide analysis in *Plasmodium falciparum* reveals early and late phases of RNA polymerase II occupancy during the infectious cycle. *BMC Genomics* 15:18. 10.1186/1471-2164-15-959 25373614 PMC4232647

[B31] Schaeffer D. Tsanova B. Barbas A. Reis F. P. Dastidar E. G. Sanchez-Rotunno M. (2009). The exosome contains domains with specific endoribonuclease, exoribonuclease and cytoplasmic mRNA decay activities. *Nat. Struct. Mol. Biol.* 16 56–62. 10.1038/nsmb.1528 19060898 PMC2615074

[B32] Schneider C. Kudla G. Wlotzka W. Tuck A. Tollervey D. (2012). Transcriptome-wide analysis of exosome targets. *Mol. Cell.* 48 422–433. 10.1016/j.molcel.2012.08.013 23000172 PMC3526797

[B33] Schneider C. Leung E. Brown J. Tollervey D. (2009). The N-terminal PIN domain of the exosome subunit Rrp44 harbors endonuclease activity and tethers Rrp44 to the yeast core exosome. *Nucleic Acids Res.* 37 1127–1140. 10.1093/nar/gkn1020 19129231 PMC2651783

[B34] Staals R. H. Bronkhorst A. W. Schilders G. Slomovic S. Schuster G. Heck A. J. (2010). Dis3-like 1: a novel exoribonuclease associated with the human exosome. *EMBO J.* 29 2358–2367. 10.1038/emboj.2010.122 20531389 PMC2910272

[B35] Tarasov A. Vilella A. J. Cuppen E. Nijman I. J. Prins P. (2015). Sambamba: fast processing of NGS alignment formats. *Bioinformatics* 31 2032–2034. 10.1093/bioinformatics/btv098 25697820 PMC4765878

[B36] Tomecki R. Kristiansen M. S. Lykke-Andersen S. Chlebowski A. Larsen K. M. Szczesny R. J. (2010). The human core exosome interacts with differentially localized processive RNases: hDIS3 and hDIS3L. *EMBO J.* 29 2342–2357. 10.1038/emboj.2010.121 20531386 PMC2910271

[B37] Ule J. Jensen K. Mele A. Darnell R. B. (2005). CLIP: a method for identifying protein-RNA interaction sites in living cells. *Methods* 37 376–386. 10.1016/j.ymeth.2005.07.018 16314267

[B38] Ule J. Jensen K. B. Ruggiu M. Mele A. Ule A. Darnell R. B. (2003). CLIP identifies nova-regulated RNA networks in the brain. *Science* 302 1212–1215. 10.1126/science.1090095 14615540

[B39] Vembar S. S. Droll D. Scherf A. (2016). Translational regulation in blood stages of the malaria parasite *Plasmodium spp*.: systems-wide studies pave the way. *Wiley Interdiscip. Rev. RNA* 7 772–792. 10.1002/wrna.1365 27230797 PMC5111744

[B40] World Health Organization [WHO] (2018). *World Malaria Report.* Geneva: WHO Press.

[B41] Zhang Q. Siegel T. N. Martins R. M. Wang F. Cao J. Gao Q. (2014). Exonuclease-mediated degradation of nascent RNA silences genes linked to severe malaria. *Nature* 513 431–435. 10.1038/nature13468 25043062

