# Supplementary figures and images for: TRIBE Uncovers the Role of Dis3 in Shaping the Dynamic Transcriptome in Malaria Parasites
Source: Front Cell Dev Biol. 2019 Nov 1;7:264. doi: 10.3389/fcell.2019.00264 (PMC6838019; doi:10.3389/fcell.2019.00264)

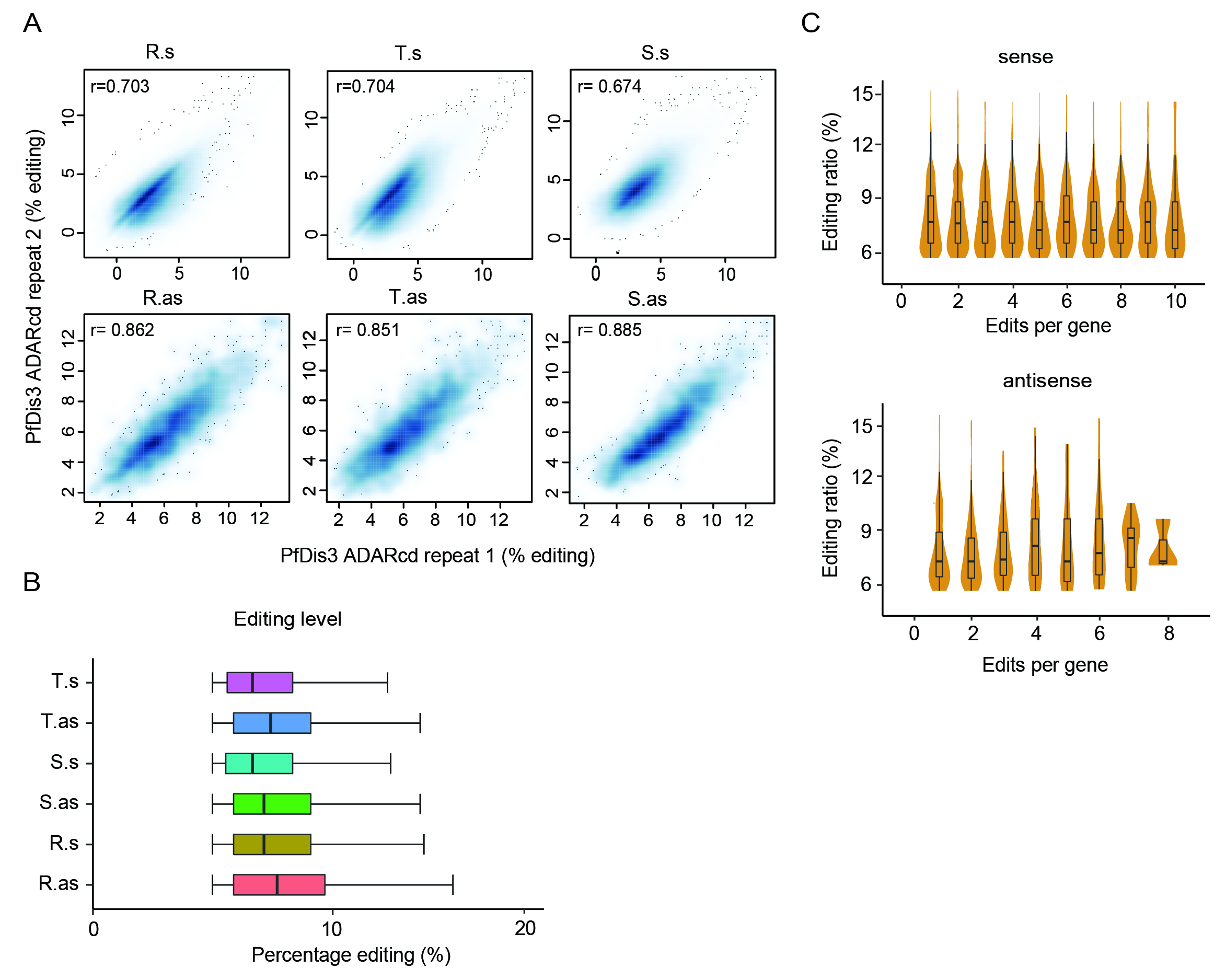

Supplement: FIGURE S1 — The PfDis3-ADARcd reproducibly edits certain sites in P.falciparum during the IDC. (A) The edit sites at sense (top) transcripts and antisense (bottom) transcripts are reproducible in editing frequency at ring (R), trophozoite (T) and schizont (S) stages. Pearson correlation between biological replicates are shown. (B) Box plot showing the PfDis3-ADARcd editing levels in sense (s) transcripts and antisense (as) transcripts at ring (R), trophozoite (T) and schizont (S) stages. (C) Box plots showing the editing frequency of sense transcripts and antisense transcripts grouped by the number of edit sites. The middle line in the box indicates the mean of the editing frequency in the transcripts with more than one edit sites. [file Image_1.JPEG]

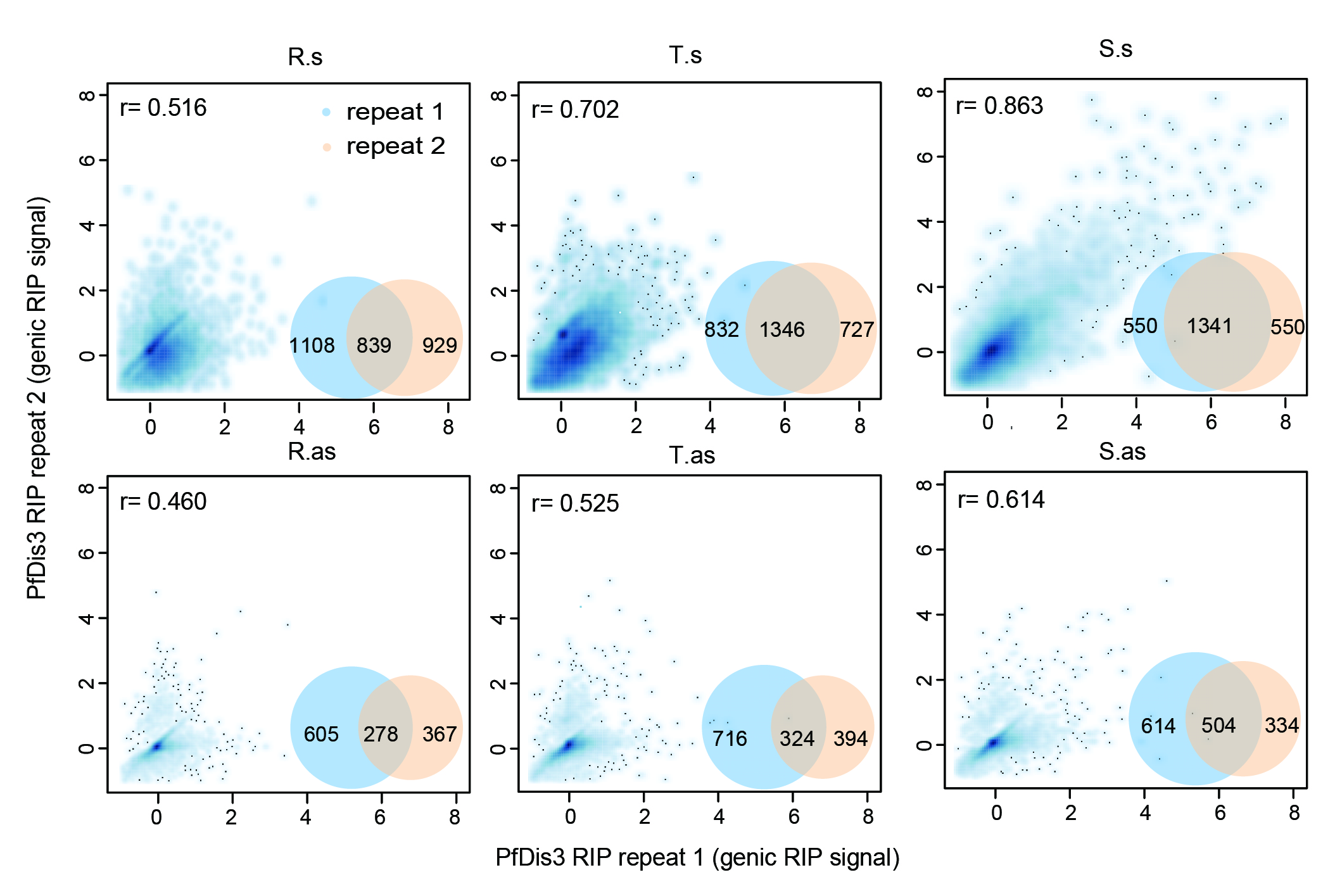

Supplement: FIGURE S2 — Reproducibility of PfDis3-RIP assay across developmental stages. Correlation of genic RIP signals in sense (s) transcripts and antisense (as) transcripts between biological replicates. Pearson correlation coefficients between biological replicates are displayed at top left corner. The inset Venn diagram showing the overlap of PfDis3 targets identified by PfDis3-RIP between the two replicates. R, T, S indicates Ring, Trophozoite and Schizont stage, respectively. [file Image_2.jpg]

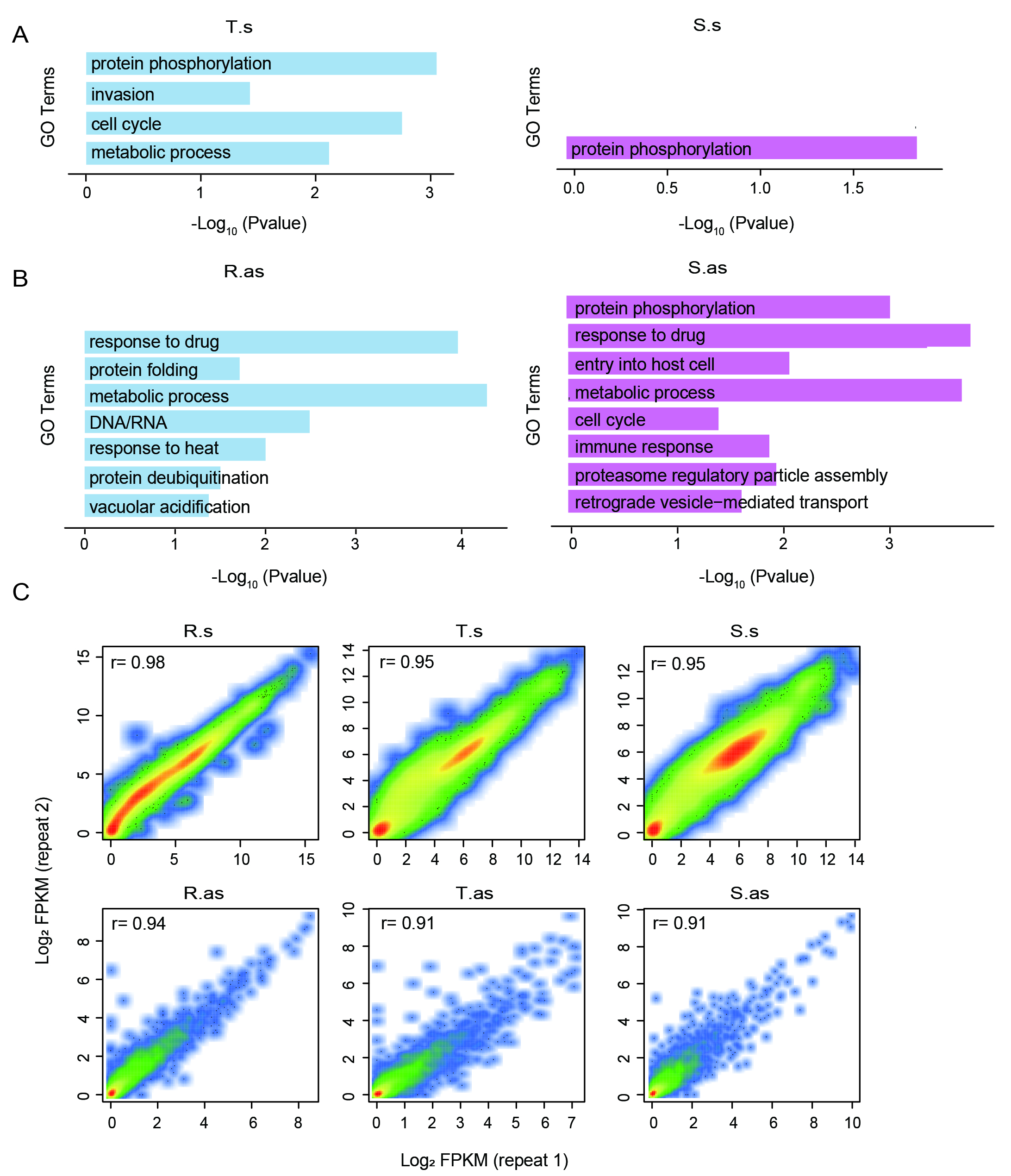

Supplement: FIGURE S3 — Functions of PfDis3-TRIBE target genes during the IDC in P. falciparum. (A) Enriched Gene Ontology (biological processes) terms for PfDis3 sense target transcripts at T (left panel) and S (right panel) stages, respectively. (B) Enriched GO terms for PfDis3 antisense target transcripts at ring R (left panel) and S (right panel) stages, respectively. (C) Correlation for expression of sense (top) transcripts and antisense (bottom) transcripts between biological replicates. Pearson correlation coefficients between biological replicates are displayed at top left corner. R, T and S indicates Ring, Trophozoite, Schizont stage, respectively. [file Image_3.JPEG]
